# A RGB-Type Quantum Dot-based Sensor Array for Sensitive Visual Detection of Trace Formaldehyde in Air

**DOI:** 10.1038/srep36794

**Published:** 2016-11-10

**Authors:** Hui Xia, Jing Hu, Jie Tang, Kailai Xu, Xiandeng Hou, Peng Wu

**Affiliations:** 1 College of Chemistry, Sichuan University, Chengdu 610064, China; 2 Analytical & Testing Center, Sichuan University, Chengdu 610064, China

## Abstract

A simple colorimetric sensor array based on red-emitting CdTe QDs and green-colored fluorescein that exhibited RGB-type color change was proposed for visual detection of trace formaldehyde. In the presence of formaldehyde, the red fluorescence from CdTe QDs was quenched while the green fluorescein was inert thus as a reference. Through harvesting the varied quenching efficiency of different ligand-capped CdTe QDs by formaldehyde, a simple sensor array can be constructed for both selective detection of formaldehyde with high sensitivity (LOD of 0.08 ppm) and identification of the existence of potential interference from acetaldehyde. The quenching mechanisms of formaldehyde toward different ligand capped CdTe QDs were studied with fluorescence lifetime, zeta potential, and also theoretical calculations. The results from theoretical calculations were in good agreement with the experimental results. The proposed sensor array was successfully explored for visual analysis of formaldehyde in indoor air samples.

Quantum dots (QDs) have received great attention in sensor applications, luminescent biolabels, and bioimaging probes^1^,^2^,^3^,^4^. It is noteworthy that the optical properties of QDs are very sensitive to their surface states, and this is essentially the basis of a variety of QD-based probes for small molecules and ions^5^,^6^,^7^,^8^. In particular, the surface ligands anchored during synthesis or water-solubilization of the QDs could vary the selectivity toward analytes^9^,^10^,^11^, thus providing more versatility in sensor fabrication over conventional organic dyes. As to specific-functionalized QDs for analysis of biomolecules (e.g., DNA and proteins), the variation of QD fluorescence caused by the change of artificial surface states should be minimized to increase the accuracy of the analyte readout^2^,^12^. As a result, it is possible to develop new sensor arrays making use of the fact that small molecules are very sensitive to the surface states/changes of QDs^13^,^14^,^15^,^16^.

RGB (red, green and blue)-based color change has been extensively explored in sensor development since the analyte response can be directly and sensitively read out with naked eye^17^,^18^,^19^. For example, Suslick and co-workers have established a series of organic dyes-based sensor arrays for discriminating analytes with the RGB values of the difference maps^20^,^21^. Particularly, ratiometric sensing with “traffic light”-type response, i.e., encoding the signal with one color as the signal code and another as the reference, is a typical application of RGB for visual analysis^22^,^23^. In this manner, the facile tunability of QD fluorescence holds great opportunity for RGB-based visual analysis^24^,^25^,^26^,^27^.

Herein, we proposed a simple RGB-type sensor array based on color modulation of different ligands-capped CdTe QDs. Formaldehyde was selected as a model analyte, which has been discussed as a typical indoor pollutant for decades^28^,^29^. Traditional techniques used for formaldehyde detection are based on gas chromatograph, mass spectroscopy, fourier transform infrared spectroscopy (FTIR), ultraviolet-visible spectrometry (UV/Vis) and fluorimetry^28^,^29^, which are inconvenient for on-site direct visual readout. To realize the RGB color profiling, red-emitting CdTe QDs were obtained from aqueous synthesis^30^, and green-colored fluorescein was introduced. The red fluorescence of QDs could be quenched by formaldehyde because of possible reactions between formaldehyde and the chosen ligands. Meanwhile, the green color from fluorescein is inert to formaldehyde and thus can be explored as the reference. Accordingly, the presence of formaldehyde yielded ratiometric responses, which provided a built-in correction for the environmental effect that may affect fluorescent measurements. Moreover, the fluorescent color changed from red to green is sensitive to naked eye. Based on the cross-reactivity generated by different ligand-capped QDs, therefore, a simple sensor array for formaldehyde was developed. Hence, reading out of formaldehyde can be simply achieved via color change of the sensor array.

## Results

### Design of a ratiometric probe for formaldehyde

Here we used a simple QD-dye mixed system to develop the ratiometric visual sensing moiety, since the broad absorption of QDs provides vast potential for simultaneous excitation of both QDs and dyes^31^. CdTe QDs were selected since they can be facilely synthesized via aqueous synthetic routes with high fluorescent yield^30^. To achieve visual color profiling, red-emitting CdTe QDs (with six common thiols as capping ligands: three amino acid L-Cys, N-A-cys, GSH, and three carboxylic acid, TGA, MPA and MSA) were obtained with a one-step refluxing route^32^. The UV-Vis absorption and photoluminescence (PL) spectra of L-Cys capped CdTe QDs were given in Figure S1A (the rest were given in Figure S2 and Table S1). A well-resolved absorption maximum of the first electronic transition at 595 nm and a red PL emission band of 630 nm were identified for L-Cys capped CdTe QDs (Figure S1A). To supply a reference color that facilitates visual readout, green-emitting fluorescein was chosen for use, which exhibits a bright emission band at 530 nm. After mixing, the PL spectra of the CdTe QDs-fluorescein mixture retain the profile of both CdTe QDs and fluorescein (Figure S1B). Importantly, both CdTe QDs and fluorescein can be excited at 365 nm (Figure S1B), which paves the way of using a handheld UV lamp for convenient visual detection. The variation of fluorescent color between red and green is advantageous since both red and green are among the most sensitive colors to human eyes^24^,^25^,^26^,^33^.

**Figure 1:**
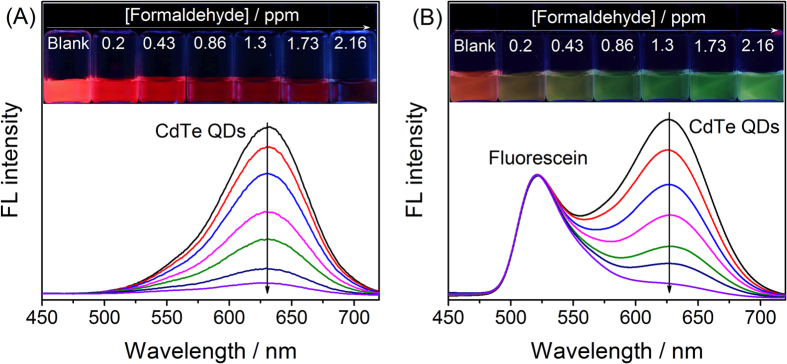
The PL spectra of L-Cys capped QDs (**A**) and L-Cys capped QDs- fluorescein mixed solution (**B**) in the presence of increasing concentrations of formaldehyde. Inset: photos showing the fluorescence color change of the corresponding solutions under 365 nm UV light.

In the presence of formaldehyde, the PL of all six kinds of ligand-capped CdTe QDs were continuously quenched in seconds (less than 10 s, Figure S3). Take L-Cys capped CdTe QDs as an example, the fluorescent photos of pure L-Cys capped CdTe QDs in the presence of varied concentrations of formaldehyde were shown in Fig. 1A. However, it was difficult to differentiate the slight color changes by naked eye. For other thiols-capped CdTe QDs, the situations were similar to that of L-Cys capped CdTe QDs. In contrast, the fluorescence color of the CdTe QDs-fluorescein mixed solution changed continuously as demonstrated in Fig. 1B. Clearly, even a slight decrease of the emission intensity at 630 nm could result in distinguishable color change from the background. Accordingly, the mixture of CdTe QDs-fluorescein can be explored as ratiometric probes for formaldehyde with RGB-type visual read out.

**Figure 2:**
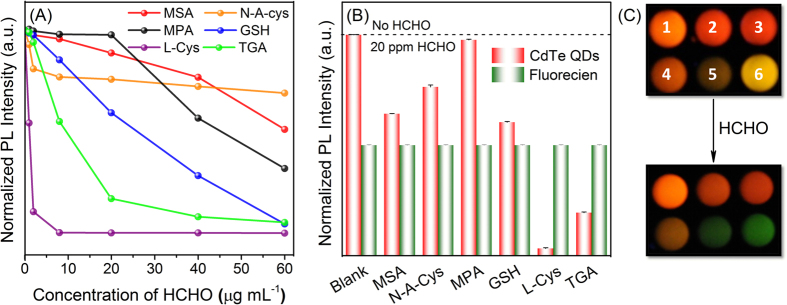
PL response of each ligand capped CdTe QDs (**A**) versus increasing formaldehyde concentrations; (**B**) after exposure to 20 ppm formaldehyde; and (**C**) a typical graph of visual detection of formaldehyde with the CdTe QDs array under 365 nm UV light (1-6: MSA-, N-A-cys-, MPA-, GSH-, L-Cys-, and TGA-capped CdTe QDs, respectively).

Due to different functional groups of the six thiols-capped CdTe QDs, different PL quenching efficiencies by increasing concentrations of formaldehyde on the QDs were observed (Fig. 2A), in which L-Cys and TGA capped-CdTe QDs received larger quenching efficiency by formaldehyde than other CdTe QDs. The Occupational Safety and Health Administration (OSHA) has set the immediately dangerous to life or health (IDLH) limit for formaldehyde at 20 ppm^34^. With such a formaldehyde concentration, the PL intensity of fluorescein was stable (Fig. 2B, and is also stable in the range of 0–30 ppm), but for the six CdTe QDs, the PL intensity was quenched more or less. Still, L-Cys- and TGA-capped CdTe QDs received the largest quenching efficiencies, accompanied by fluorescent color change from yellow/orange to green (Fig. 2C). For the rest MSA-, N-A-cys-, MPA- and GSH-capped CdTe QDs, relatively slight PL intensity change was observed (Fig. 2B), with varied but overall minimal fluorescent color change (Fig. 2C). The quenching mechanism will be discussed later in the section of “Mechanistic study”. Therefore, all these ratiometric systems were explored together to develop a RGB-type sensor array for formaldehyde.

### Formaldehyde detection

Since L-Cys-capped CdTe QDs received the largest quenching efficiency by formaldehyde than other CdTe QDs, it was explored for further formaldehyde quantification. The linear relationship between the PL intensity of L-Cys-capped CdTe QDs and the formaldehyde concentration was illustrated in Figure S4. The limit of detection (LOD, 3σ) is evaluated as 0.08 ppm, which is the same as that for safe-exposure standard set by World Health Organization (WHO)^35^, and is about 10 times lower than permissible exposure limit (750 ppb) by OSHA^34^. The PL spectra of CdTe QDs-fluorescein mixture in the presence of formaldehyde and the corresponding calibration curves for other five ligand-capped CdTe QDs were summarized in Figures S5–S9 and Table S2.

Quantitative formaldehyde analysis could also be simply performed with the proposed sensor array (six ligands-capped CdTe QDs-fluorescein systems) base on calculation of the total Euclidean distance (*d*_*mn*_) of the array (Figure S10)^21^,^36^. The Euclidean distance is simply evaluated based on the total length of intensities of 6 ligands capped CdTe QDs for varied formaldehyde concentrations (5 replicates). As shown in Figure S10, by using the total Euclidean distance as an overall measure of the array response, one can also generate monotonic (but obviously not linear) response curves with increasing formaldehyde concentration from 0 to 30 ppm. The lowest quantification level for formaldehyde by Euclidean distance evaluation was similar to that obtained with L-Cys-capped CdTe QDs (0.08 ppm).

### Selectivity of CdTe QDs array for formaldehyde detection

**Figure 3:**
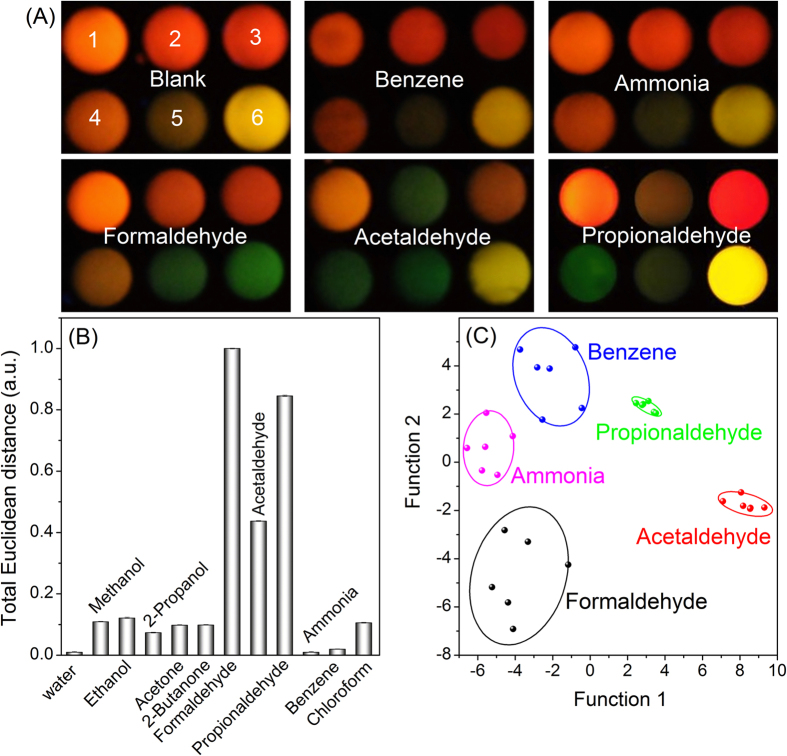
Selectivity of CdTe QDs array for formaldehyde detection: (**A**) color change patterns of main co-existing indoor gases (1-6: MSA, N-A-cys, MPA, GSH, L-Cys, and TGA-capped CdTe QDs, respectively); (**B**) interference with formaldehyde (5 ppm) determination at 50 ppm of interference reagents, the total Euclidean distance was evaluated by the method showing in Figure S10; and (**C**) LDA cluster analysis for distinguishing main co-existing indoor gases.

In real indoor sample analysis, formaldehyde often co-exists with other volatile pollutants, such as acetaldehyde, benzene, and ammonia (Table S3)^37^, but many of them (such as toluene and xylenes) have extremely low solubility in water. Therefore, they are majorly excluded during the sampling stage and thus their potential interferences are not considered in this work. Accordingly, the selectivity of the proposed array was assessed by challenging the above possible interfering species. As shown in Fig. 3A, compared with the blank, benzene (the example for insoluble VOCs) and ammonia caused little change to the fluorescence color of the array. For acetaldehyde and propionaldehyde, potential interference was observed, since they also induced PL quenching of N-A-cys-, L-Cys-, GSH- and TGA- capped CdTe QDs. The extraction of the total Euclidean distances also confirmed such interferences (Fig. 3B). However, linear discriminant analysis (LDA) dendrogram showed that formaldehyde, acetaldehyde, propionaldehyde, benzene and ammonia could be distinguished from each other (10 ppm) in 95% confidence interval (Fig. 3C). Other potential co-existing water-soluble VOCs, such as alcohols, ketones and chloroform, caused minimal interferences with formaldehyde (5 ppm) detection at their concentration level of 50 ppm (Fig. 3B).

**Figure 4:**
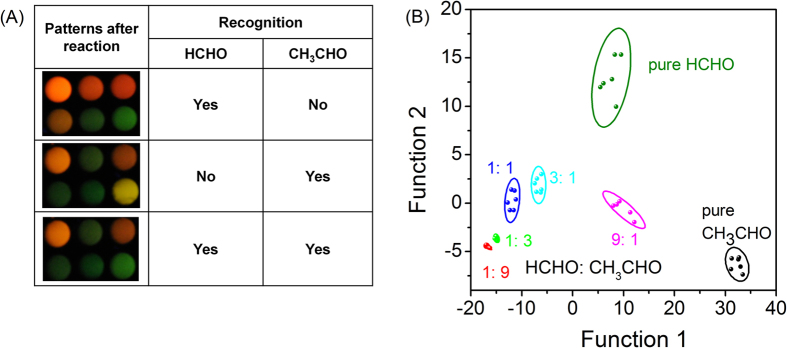
Strategy for analysis of samples containing both formaldehyde and acetaldehyde. (**A**) visual differentiation of samples containing both formaldehyde and acetaldehyde; and (**B**) LDA cluster analysis for distinguishing different ratios of formaldehyde to acetaldehyde (total concentration of formaldehyde and acetaldehyde: 20 ppm, the ratios were acetaldehyde/formaldehyde: v/v).

In real sample analysis of formaldehyde with the conventional sensor (single sensor element), the potential interference from acetaldehyde and propionaldehyde may be problematic since all three can quench the PL of QDs. However, such problem can be simply alleviated to a large extent with the proposed sensor array. First, samples containing only acetaldehyde, or formaldehyde, or both, can be distinguished with the array pattern (Fig. 4A. Here, we majorly considered the interference from acetaldehyde since propionaldehyde caused less serious interference as compared to acetaldehyde. If both existed, they can be approximately treated as the same.). Next, a series of acetaldehyde and formaldehyde mixtures with different concentration ratios were prepared and subjected to array analysis. The standard array response pattern of the mixtures was generated through LDA clustering, several proportions of formaldehyde and acetaldehyde could be easily distinguished (Fig. 4B). Although MPA- and MSA-capped CdTe QDs played less significant roles in visual discrimination of acetaldehyde and formaldehyde (Fig. 4A), they were indispensable in generating the standard array response pattern for further identification (Figure S11). For unknown samples, first they were also analyzed with the sensor array and clustered. In this manner, the rough ratios of acetaldehyde and formaldehyde in the samples can be evaluated. Next, the formaldehyde concentration can be estimated mathematically based on the calibration data of formaldehyde (see the following section for details). It should be noted that with the conventional selective sensors, potential interferences cannot be predicted. However, with the proposed sensor array, interference from acetaldehyde can be directly visualized and differentiated.

### Real indoor sample analysis

**Figure 5:**
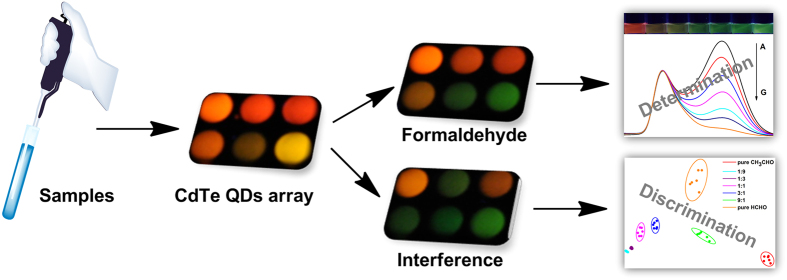
Schematic illustration of the process for sample analysis and discrimination.

**Figure 6:**
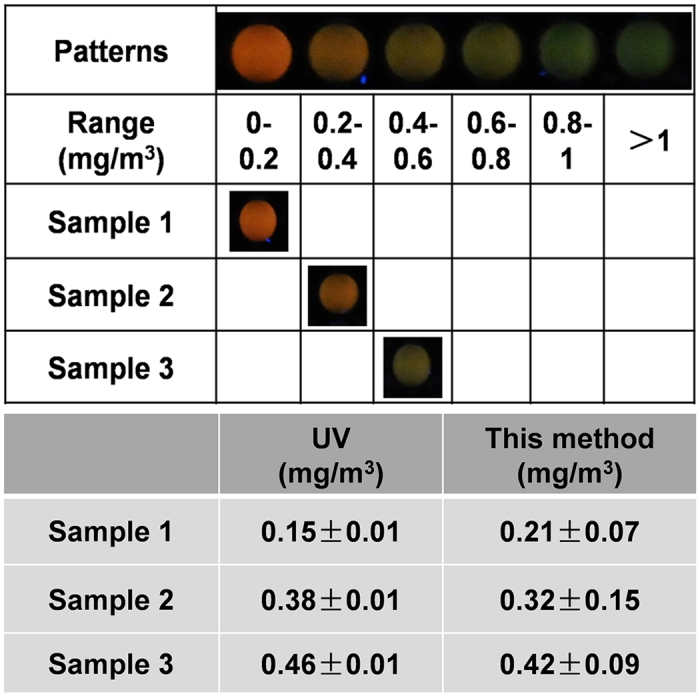
Visual analysis of real indoor air samples with the proposed sensor array.

On the basis of the above discussions, the process for visual detection of formaldehyde in real samples was summarized in Fig. 5. First, the samples are exposed to the sensor array to identify whether acetaldehyde is presented. If color changes only generate on L-Cys- and TGA-capped CdTe QDs, only formaldehyde is present in the sample (little acetaldehyde). As a result, determination of formaldehyde in the samples could be obtained by PL evaluation of either L-Cys- or TGA-capped CdTe QDs directly. Otherwise the presence of acetaldehyde in the sample can be verified through fluorescent color change of N-A-cys-, GSH- and L-Cys capped CdTe QDs. In this manner, the rough ratios of formaldehyde and acetaldehyde can be evaluated through LDA clustering. Next, the concentrations of formaldehyde and acetaldehyde could be obtained by PL change of TGA- capped CdTe QDs and N-A-cys- or GSH- capped CdTe QDs, respectively. To test the feasibility of the proposed sensor array for analysis of real samples for formaldehyde, five indoor air samples were collected from local furniture markets and analyzed with the sensor array. Besides, two certified reference samples (GBW (E) 081701 and BW 3450) were analyzed. The analytical results were summarized in Tables S4 and S5, which were in good agreement with those by a standard method or certified values. A *t*-test shows that the analytical results by the proposed method have no significant difference from the certified values at the confidence level of 95%. Further, the visual analysis of real samples was also easily achieved (Fig. 6 and Table S6).

## Discussion

**Figure 7:**
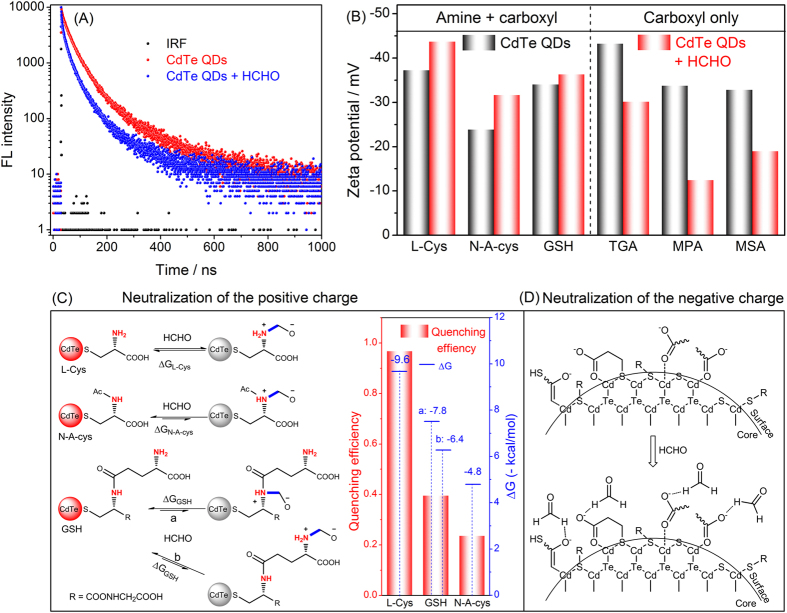
Mechanistic study about the formaldehyde-induced quenching toward CdTe QDs. (**A**) fluorescence lifetime of L-Cys-capped CdTe QDs in the absence and presence of formaldehyde; (**B**) zeta potential investigations on CdTe QDs in the absence and presence of formaldehyde; (**C**) possible interaction pathways for formaldehyde with L-Cys, N-A-cys, and GSH-capped CdTe QDs, the inset shows the relationship between quenching efficiency and the ΔG of the possible quenching reactions (obtained from theoretical calculations); and (**D**) possible interaction pathways for formaldehyde with TGA, MPA, and MSA-capped CdTe QDs.

In this work, different quenching effects of formaldehyde on the PL of the six thiols-capped CdTe QDs were observed, and this is the basis for construction of the sensor array. Fluorescence lifetime analysis showed that the lifetime of all CdTe QDs was considerably shortened in the presence of formaldehyde (Fig. 7A and Figure S12), indicating dynamic quenching by formaldehyde. There is no spectral overlap between the emission of CdTe QDs and the absorption of formaldehyde, probably energy transfer between the QDs and formaldehyde can be ruled out. Moreover, since thiol groups possess higher affinity with CdTe QDs than formaldehyde, possible ligand displacement caused quenching can also be excluded. Therefore, possible electron transfer may be the major contribution for the observed quenching.

For amino acid (or peptide)-capped QDs (L-Cys, N-A-cys, and GSH), it can be expected that nucleophilic addition reactions from amine to carbonyl of formaldehyde would occur, which can be verified from the zeta potential change of these QDs in the presence of formaldehyde (Fig. 7B). The possible pathways for such nucleophilic addition reactions were given in Fig. 7C, and the change of Gibbs free energies at 298 K (ΔG_298_) corresponding to each reaction was evaluated with theoretical calculations (see the Supplementary Information for details). As shown in the inset in Fig. 7C, the ΔG_298_ values of all calculated reactions are negative, suggesting that these nucleophilic addition reactions should be thermodynamically favorable. Interestingly, the degree of fluorescence quenching is likely to be affected by the extent of the nucleophilic addition reactions. The absolute values of ΔG_298_ of the corresponding quenching reactions correlate well with the experimental quenching efficiencies of formaldehyde to these ligands capped CdTe QDs, for both ΔG_298_ and quenching efficiency, L-Cys > GSH > N-A-cys. The nucleophilicity of primary amine in L-Cys with formaldehyde is stronger than those of secondary amine groups in N-A-cys and GSH, therefore, thermodynamically more favorable with the nucleophilic addition reaction and thus the best quenching.

For TGA, MPA, and MSA-capped CdTe QDs (only carboxyl), the zeta potential changes are in contrast to those amino acid-capped QDs (Fig. 7B). It’s well-known that the carboxyl group as well as the branched conditions of capping ligands play an important role to the luminescence efficiency of QDs^38^,^39^. Considering the surface structure of carboxyl-terminated QDs^38^, the presence of formaldehyde may compete with the surface carboxyl groups. Therefore, the zeta potentials of these QDs should move to positive, since negative charge is alleviated via hydrogen bonding between hydrogen in formaldehyde and oxygen from the surface hydroxyl groups (Fig. 7D), leading to surface state change of these QDs and fluorescence quenching. TGA-capped CdTe QDs received heavier quenching than MPA-capped ones by formaldehyde, probably because the surface carboxyl groups in TGA-capped QDs are closer to QDs than those in MPA-capped ones. Since MSA possesses branched structure, it is expected that competition of surface carboxyl groups would result in less perturbation of surface states. Therefore, MSA-capped CdTe QDs would receive less quenching than TGA- and MPA-capped CdTe QDs, which is consistent with experimental results. However, the PL quenching is a complex process that can be affected by many factors. It should be aware that the mechanism cannot be fully predicted by theoretical investigations or circumstantial evidences and requires further in-depth study.

## Conclusion

In summary, a simple and sensitive sensor array for visual detection of formaldehyde was developed. Using six different common thiols-capped CdTe QDs as quenching units and fluorescein as the color reference, the proposed method could discriminate formaldehyde with high selectivity. An LOD of 0.08 ppm by PL detection was achieved, and the color changes could be observed directly by naked eye. The possible PL quenching pathways were discussed with theoretical calculations. The proposed methodology was proved to be effective for establishing sensor arrays based on simple synthesis and assembling of QDs, and it is expected to be extended for other analytes in a similar manner.

## Experimental section

### Synthesis of CdTe QDs capped with six different ligands

Red-emitting CdTe QDs were prepared with a one-step aqueous refluxing route described previously^40^. Six common thiols, namely mercaptosuccinic acid (MSA), N-acetyl-cysteine (N-A-Cys), 3-mercaptopropionic acid (MPA), glutathione (GSH), L-cysteine (L-Cys), and thioglycolic acid (TGA), were chosen as ligands for CdTe QDs. The molar ratio of Cd^2+^: Te_2_O_3_^2-^: thiol is 5: 1: 6. Detailed synthesis was given in the Supplementary Information. The PL wavelengths of CdTe QDs used in this study are all longer than 600 nm, the final reaction time, emission wavelength, and size of each ligand capped CdTe QDs were summarised in Table S1.

### Fabrication of the sensor array

The sensor array was carried on in a 96-well microplate. Solution of six different CdTe QDs (3.80 × 10^−5^ mol/L, 150 μL) and fluorescein (1 mg/L, 150 μL) were added into the microplate, respectively. Then, different concentration of formaldehyde or samples (50 μL) were added into each well. The fluorescent color change was observed under UV excitation (365 nm).

### Sample preparation

The real samples were collected from local furniture markets. The real indoor air samples were collected with an air sampler and dissolved in ultrapure water at a flow rate of 200 mL/min for 15 min, according to the Chinese National Standards (CNS) GBZ 159. Two formaldehyde Certified Reference solution (GBW (E) 081701 and BW 3450) were diluted for 100 time with ultrapure water for further detection. All the experimental conditions and processes were the same as those for the detection of formaldehyde.

### Computational details

All the theoretical calculations were performed by DMol^3^ program in the Materials Studio package^41^,^42^, which is the quantum mechanical code using density functional theory (DFT). Perdew-Wang (1991) (PW91) function of generalized gradient approximation (GGA) level^43^ was used to calculate the geometric optimization, energy and the frontier molecular orbital. The localized double numerical polarization (DNP) basis set were applied to expand the Kohn-Sham orbitals. Self-consistent field (SCF) calculations were carried out with a convergence criterion of 10^–6^ a.u. on the total energy and the real-space global orbital cutoff was chosen to be 4.4 Å in the computations. Molecular symmetry was not enforced to allow for full geometry relaxation. The value of smearing is 0.001 Hartree. COSMO solvent calculations with the DC-PBE function were also performed using the dielectric constant of water^44^, *i.e*., 78.54.

## Additional Information

**How to cite this article**: Xia, H. *et al*. A RGB-Type Quantum Dot-based Sensor Array for Sensitive Visual Detection of Trace Formaldehyde in Air. *Sci. Rep*. **6**, 36794; doi: 10.1038/srep36794 (2016).

**Publisher’s note:** Springer Nature remains neutral with regard to jurisdictional claims in published maps and institutional affiliations.

## Supplementary Material

Supplementary Information
