# Supplementary material for: A RGB-Type Quantum Dot-based Sensor Array for Sensitive Visual Detection of Trace Formaldehyde in Air
Source: Sci Rep. 2016 Nov 10;6:36794. doi: 10.1038/srep36794 (PMC5103289; doi:10.1038/srep36794)
Supplement: Supplementary Information [file srep36794-s1.doc]

**Supplementary Information**

**A RGB-Type Quantum Dot-based Sensor Array for Sensitive Visual Detection of Trace Formaldehyde in Air**

Hui Xia,^†^ Jing Hu,^†^ Jie Tang,^†^ Kailai Xu,^†^ Xiandeng Hou,*^,†, ‡^ Peng Wu,*^,†, ‡^

**^†^**College of Chemistry, and **^‡^**Analytical & Testing Center, Sichuan University, Chengdu 610064, China.

E-mails: wupeng@scu.edu.cn; houxd@scu.edu.cn

**Experimental Section**

**1.** **Chemical Reagents.**

All reagents used in this work were of at least analytical grade without further purification. CdCl_2_·5H_2_O (99.0+%), NaBH_4_ (98.5+%), trisodium citrate dihydrate (99.0+%) from Kelong Chemical Reagent Plant (Chengdu, China); Na_2_TeO_3_ (Alfa Aesar) and glutathione (GSH, reduced, 99%), thioglycolic acid (TGA), 3-mercaptopropionic acid (MPA, 98%), mercaptosuccinic acid (MSA, 98%), N-acetyl-L-cysteine (N-A-Cys, 99%) and L-Cysteine (L-Cys, 99%) from Aladdin Chemistry Co., Ltd. (Shanghai, China) were used for synthesis of water-soluble CdTe QDs. Fluorescein (Aladdin) was used as color reference in the array system. Formaldehyde solution (37.0 - 40.0%) and other chemicals were purchased from Kelong Chemical Reagent Plant (Chengdu, China). Two formaldehyde Certified Reference solution (GBW(E)081701 and BW 3450) from the Beijing Yihua Standard Technology Co. Ltd were used to validate the accuracy of the proposed method. Ultrapure water (18.2 MΩ cm) was obtained from a water purification system (PCUJ-10, Chengdu Pure Technology Co., Ltd., Chengdu, China).

**2. Instrumentation**

Fluorescence measurements were performed on a Hitachi F-7000 PC spectro fluorophotometer (Hitachi Co., Japan) with a micro plate accessory for array establishment, the excitation wavelength was set at 365 nm during the whole process. JY02S UV lamp box for visual detection was purchased from Junyi Dongfang Electrophoresis Co., Ltd. (Beijing, China), UV-vis spectra were recorded on a UV-1750 spectrophotometer (Shimadzu Co., Japan). TY-08A intelligent air sampler (Taina Instrument Co., China) was used for collecting indoor air samples. Fluorescence lifetime measurements were performed a Fluorolog-3 spectrofluorometer (Horiba Jobin Yvon) with a Delta-diode laser (371 nm, DD-370L, Horiba Scientific) as the excitation source and a picosecond photon detection module (PPD-850, Horiba Scientific) as the detector. The reference cell contained colloidal silica, SM-30 LUDOX® solution was used as a control (Zero lifetime) for collection of instrumental response function (IRF).

**3. Synthesis of CdTe QDs**

L-Cys capped-CdTe QDs was taken as an example. Briefly, CdCl_2_·2.5H_2_O, (2.5×10^-4^ mol) was dissolved in 25 mL ultrapure water in a three-necked flask, and L-Cys (3.0 × 10^-4^ mol), trisodium citrate dihydrate, Na_2_TeO_3_ and NaBH_4_ (2.4 × 10^-4^ mol) were added and the pH was adjusted to 10.5 with 1.0 M NaOH under vigorous stirring. When the color of the solution changed to pale green, the mixture was refluxed at 100 °C and L-Cys-capped CdTe QDs began to grow immediately, longer emission wavelength and color in deeper red was observed when the refluxing time increased. All reactions were carried out under ambient atmospheric conditions. A similar procedure using TGA, MPA, GSH, MSA or N-A-Cys instead of L-Cys was applied to synthesis of different ligand capped CdTe QDs for the sensor array.

**4. Theoretical calculations**

To verify the above quenching mechanisms induced by formaldehyde, theoretical calculation was carried by employing the quantum chemical DMol^3^ approach with Materials Studio package. Taking the computational efficiency and the experimental situation into account, (CdTe)_6_ cluster was chosen for simplified model of CdTe QDs, which is also the typical structure used in previous theoretical calculations of CdTe QDs.^[S1, S2]^ The structural, energetic and electronic (the highest occupied molecular orbital and the lowest unoccupied molecular orbital, HOMO/LUMO) properties of (CdTe)_6_, (CdTe)6-L, (CdTe)_6_-L-P were investigated based on the full geometry optimizations (Table S7). Here, L represents the six different capping ligands (L-Cys, N-A-cys and GSH) used here, while (CdTe)_6_-L-P represent the products of the possible reactions between (CdTe)_6_-L and formaldehyde in Table S7, respectively. The change of Gibbs free energies at 298 K (ΔG) corresponding to each reaction was obtained from theoretical calculations and given in the inset in Figure 7C (right) and Table S8, respectively.


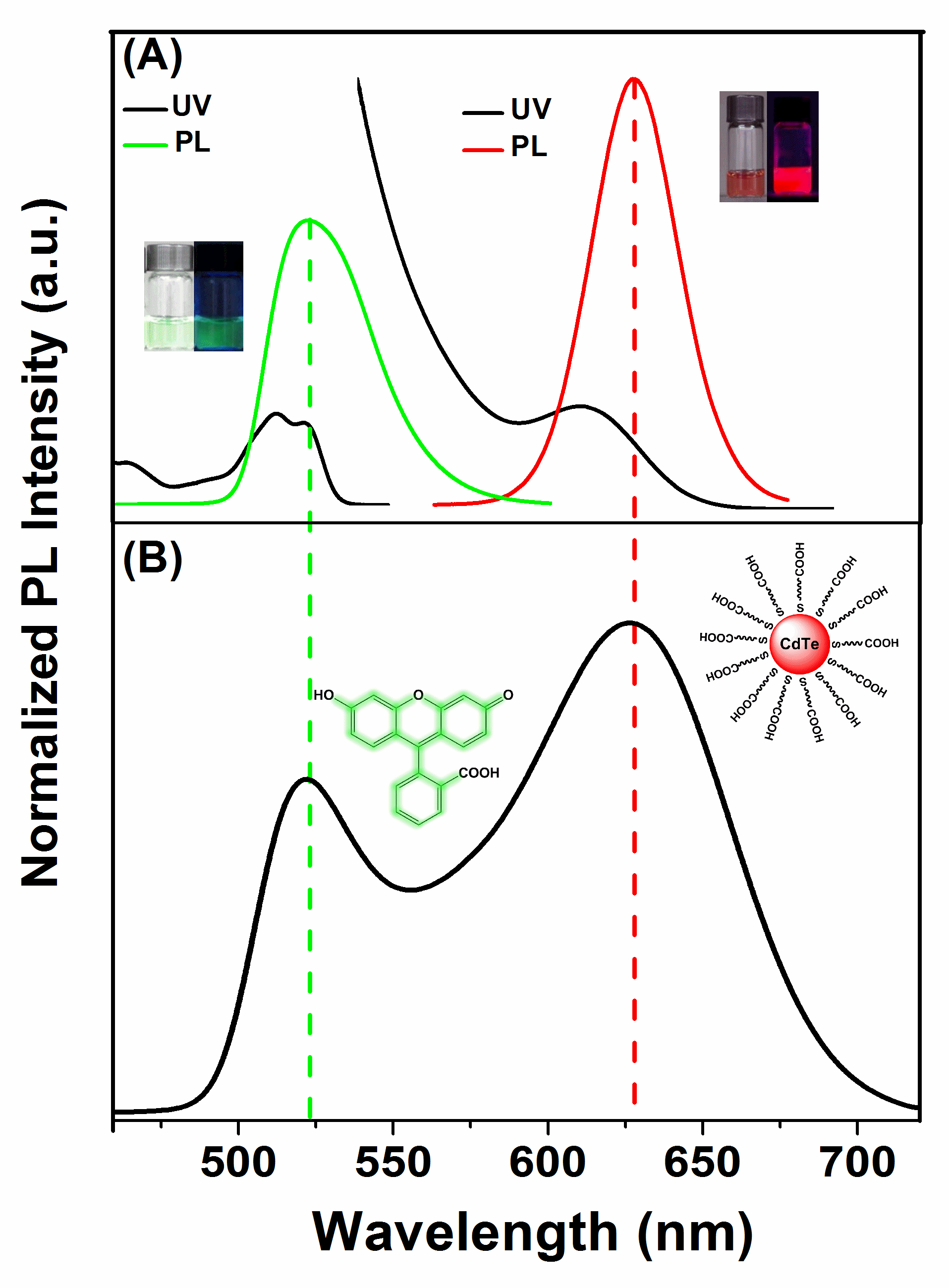


**Figure S1.** (A) UV-Vis absorption and PL spectra of L-Cys capped CdTe QDs and fluorescein, with their corresponding photographs shown in the inset; (B) PL spectra of the CdTe QDs-fluorescein mixture.


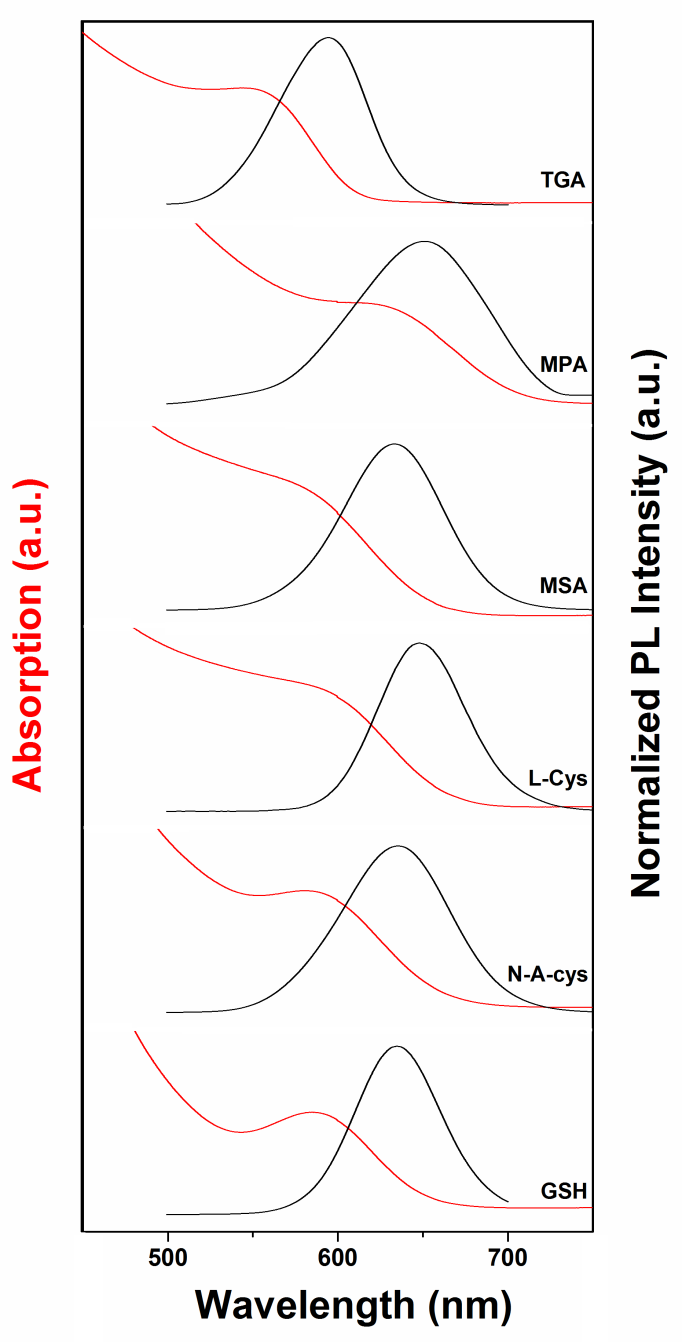


**Figure S2.** UV-vis and fluorescence spectra of six thiols-capped CdTe QDs.

**Table S1.** Reaction time, emission wavelength, and size of each ligand capped CdTe QDs.

| QDs | Reaction time (min) | λ_max_ (nm) | Core size (nm)^a^ |
| --- | --- | --- | --- |
| TGA-CdTe | 360 | 600 | 3.3 |
| MPA-CdTe | 70 | 660 | 4.0 |
| MSA-CdTe | 80 | 640 | 3.4 |
| GSH-CdTe | 80 | 640 | 3.6 |
| L-Cys-CdTe | 180 | 630 | 3.6 |
| N-A-Cys-CdTe | 360 | 645 | 3.6 |
| a: The core sizes of the 6 ligand-capped QDs were calculated based on the empirical equation proposed by Peng et al.^[S1]^ | | | |


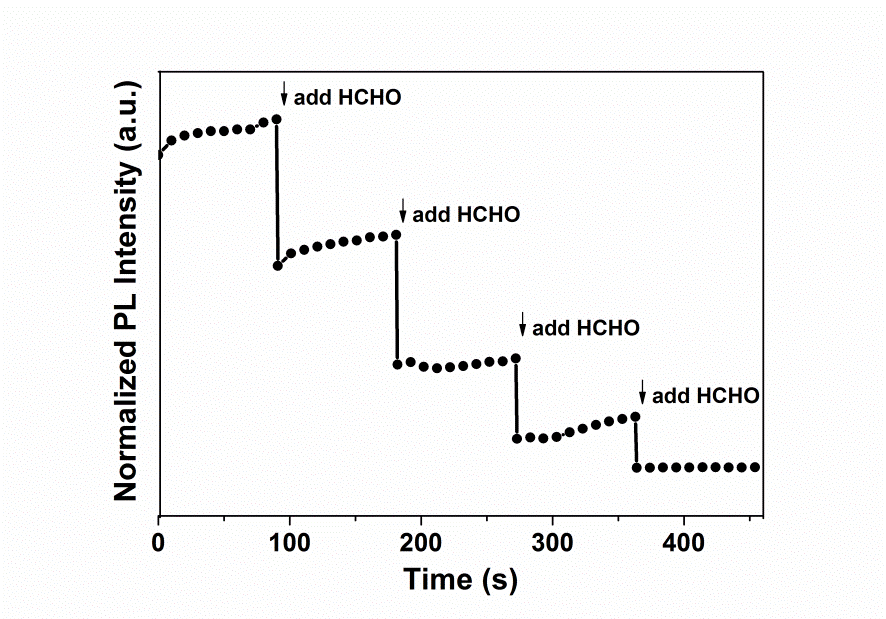


**Figure S3.** Time-course of formaldehyde-induced fluorescence quenching to L-Cys-capped CdTe QDs, the concentration of formaldehyde for every addition was 0.2 ppm.


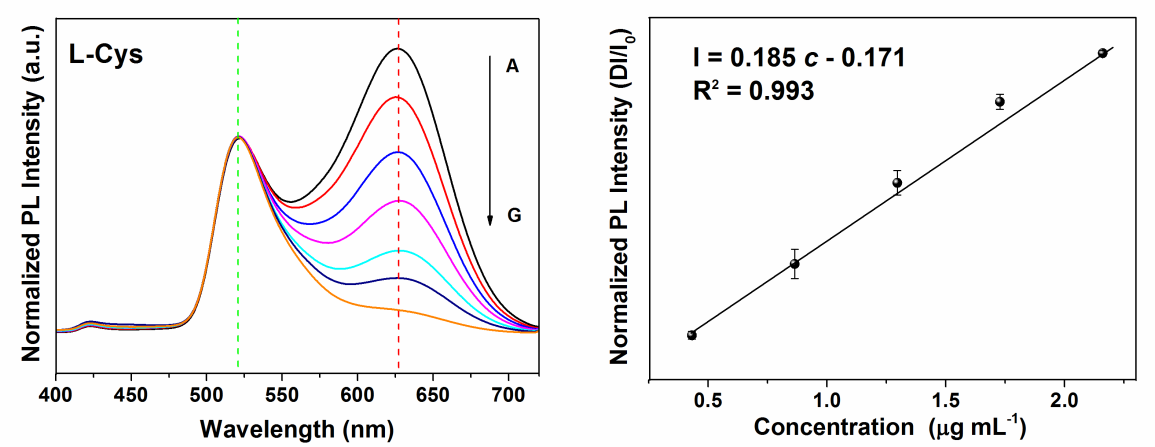


**Figure S4.** PL spectra of Q/F solution (left) and calibration curve (right) of L-Cys capped CdTe QDs in varied concentrations of formaldehyde (A, blank; B, 0.2 ppm; C, 0.43 ppm; D, 0.86 ppm; E, 1.3 ppm; F, 1.73 ppm; and G, 2.16 ppm).


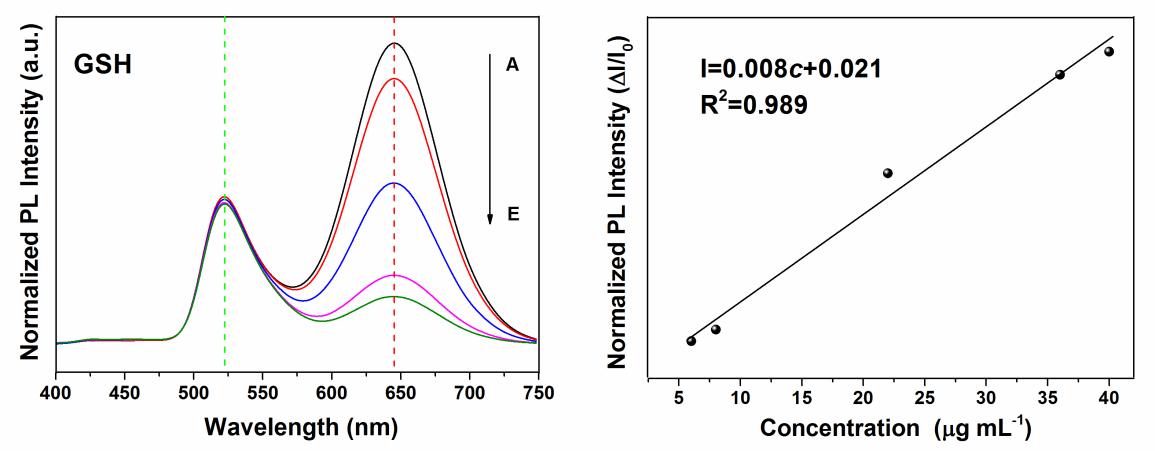


**Figure S5.** PL spectra of Q/F solution (left) and calibration curve (right) of GSH capped CdTe QDs in varied concentrations of formaldehyde (A to E: 6, 8, 22, 36, and 40 ppm).


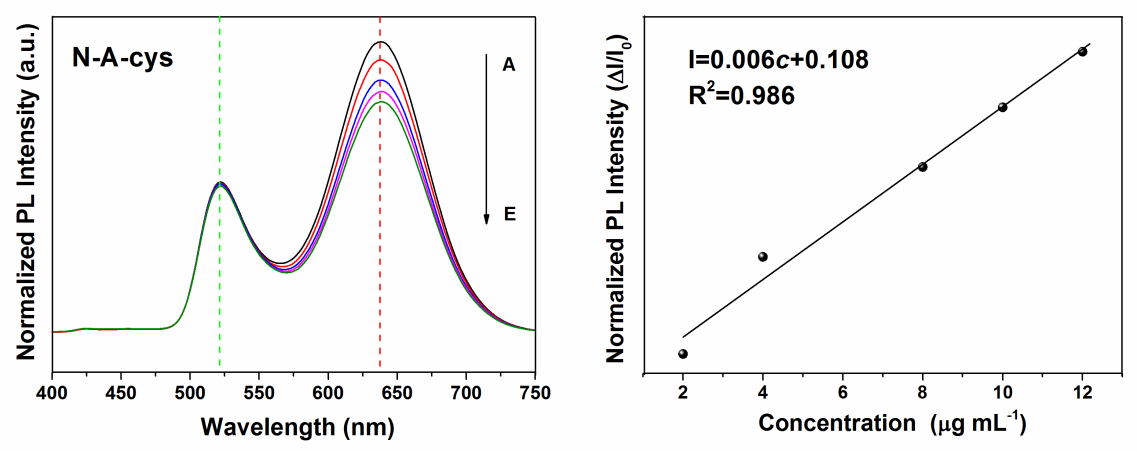


**Figure S6.** PL spectra of Q/F solution (left) and calibration curve (right) of N-A-cys capped CdTe QDs in varied concentrations of formaldehyde (A to E: 2, 4, 8, 10, and 12 ppm).


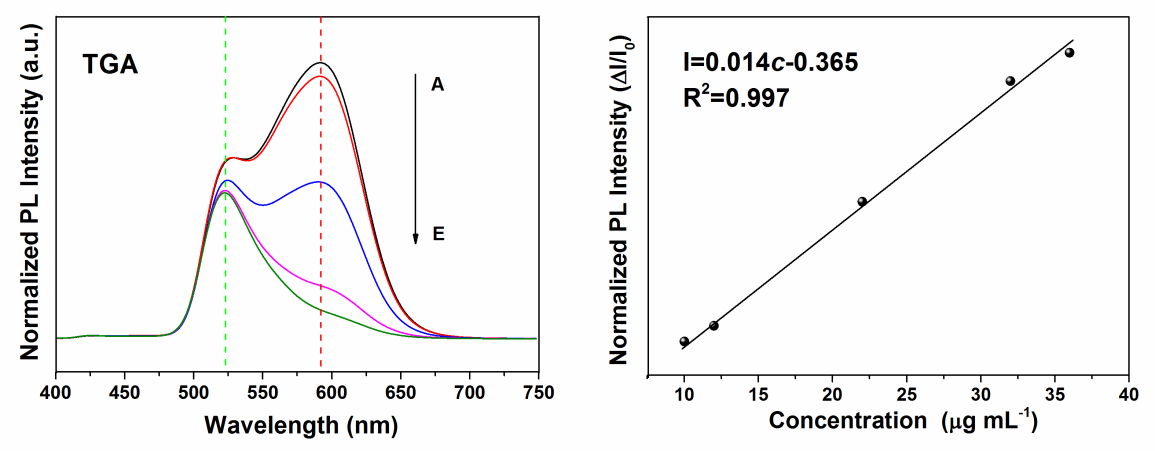


**Figure S7.** PL spectra of Q/F solution (left) and calibration curve (right) of TGA capped CdTe QDs in varied concentrations of formaldehyde (A to E: 10, 12, 22, 32, and 36 ppm).


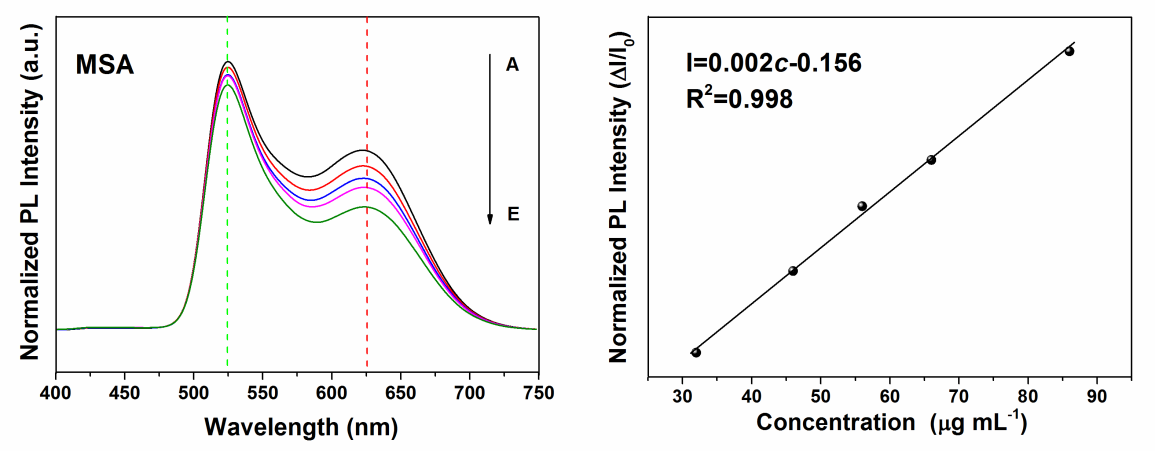


**Figure S8.** PL spectra of Q/F solution (left) and calibration curve (right) of MSA capped CdTe QDs in varied concentrations of formaldehyde (A to E: 32, 46, 56, 66, and 86 ppm).


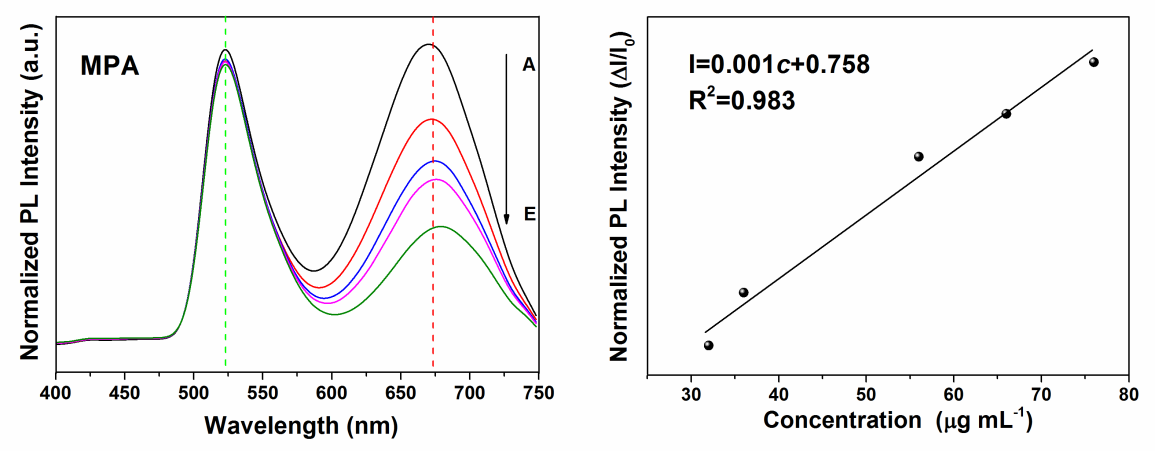


**Figure S9.** PL spectra of Q/F solution (left) and calibration curve (right) of MPA capped CdTe QDs in varied concentrations of formaldehyde (A to E: 32, 36, 56, 66, and 76 ppm).

**Table S2.** Analytical Figures of Merits of formaldehyde to 6 ligands capped CdTe QDs (*c* is the concentration of formaldehyde)

| QDs ligands | Linear equation | Linear correlation coefficient (R) | LOD (ppm) |
| --- | --- | --- | --- |
| MSA | I = 0.002*c* - 0.156 | 0.999 | 4 |
| N-A-cys | I = 0.006*c* + 0.108 | 0.993 | 2 |
| MPA | I = 0.001*c* + 0.758 | 0.991 | 20 |
| GSH | I = 0.008*c* + 0.021 | 0.994 | 4 |
| L-Cys | I = 0.185*c* - 0.171 | 0.996 | 0.08 |
| TGA | I = 0.014*c* - 0.365 | 0.998 | 2 |


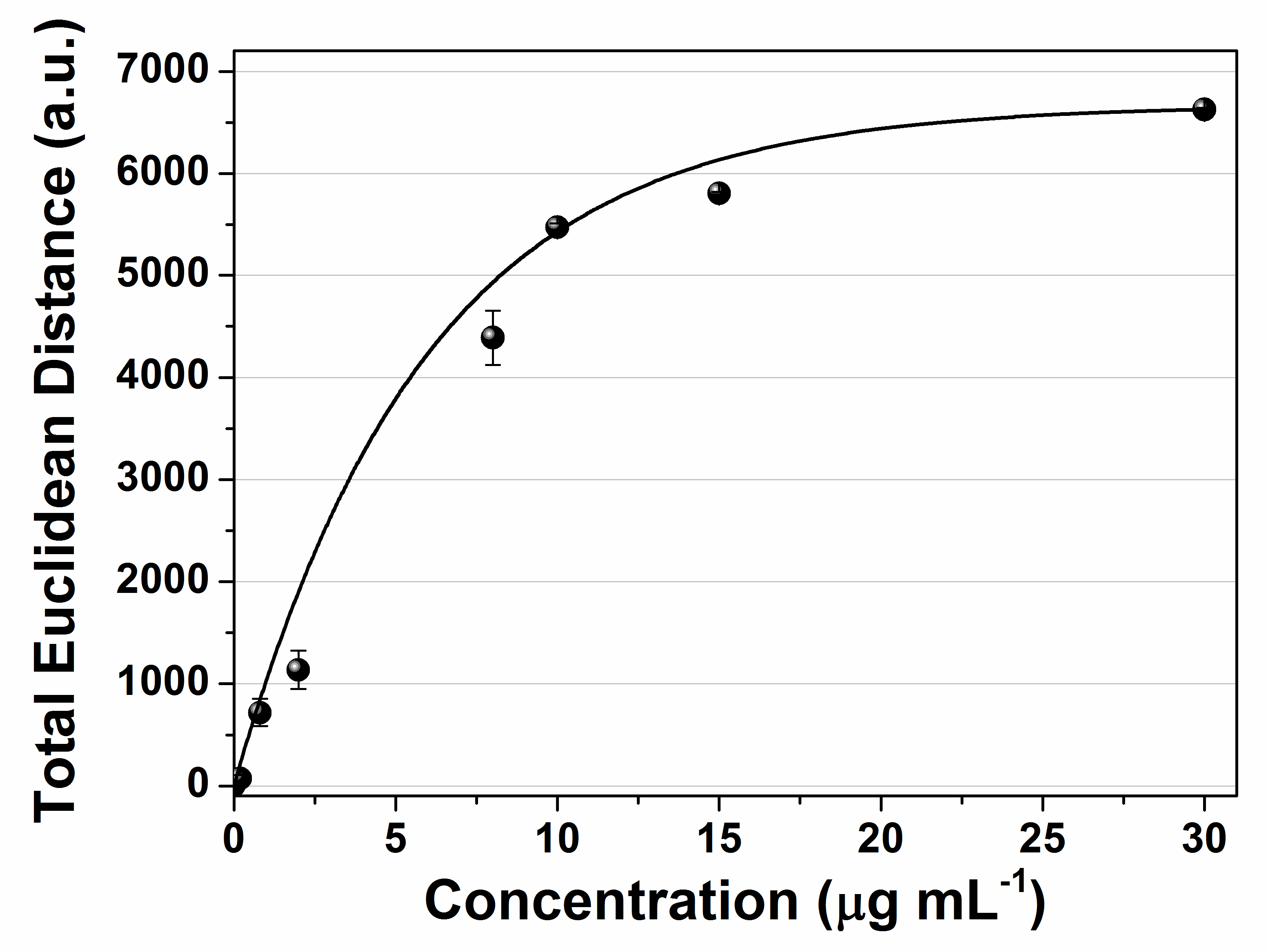


**Figure S10.** The total Euclidean distance of the PL spectra of the array versus formaldehyde concentration. *d_mn_=*△I_n_ = [(A_n_- A_0_)^2^ + (B_n_- B_0_)^2^ + (C_n_- C_0_)^2^ + (D_n_- D_0_)^2^ + (E_n_- E_0_)^2^ + (F_n_- F_0_)^2^]^1/2^, (△I_n_: Intensity change of CdTe QDs before and after reacting with formaldehyde, A-F: PL intensity of MSA, N-A-cys, MPA, GSH, L-Cys, and TGA-capped CdTe QDs, respectively).

Table S3. Common VOCs in indoor air (Information from Wang S.B., Volatile organic compounds in indoor environment and photocatalytic oxidation: State of the art. *Environ. Int.* 2007 **33**, 694-705.).

| **Chemical** | **Water solubility** | **Public places** |
| --- | --- | --- |
| Acetaldehyde | Yes | Aircraft, Office |
| Acetone | Yes | All transportations, Residential |
| Benzene | No | All transportations, Residential, Office |
| 2-Butanone | Yes | All transportations |
| Ethanol | Yes | All transportations, Residential |
| Formaldehyde | Yes | Aircraft, Train, Office |
| n-Hexane | No | Train, Bus, Subway, Office |
| Limonene | No | All transportations, Residential, Office |
| Methylene chloride | Slight, 20g/L | Aircraft, Office |
| Naphthalene | No | Aircraft, Office |
| 2-propanol | Yes | All transportations |
| Propionaldehyde | Yes | Aircraft |
| Tetrachloroethylene | No | Aircraft, Residential, Office |
| Toluene | Very slight | All transportations, Residential, Office |
| Xylenes | No | Aircraft, Residential, Office |


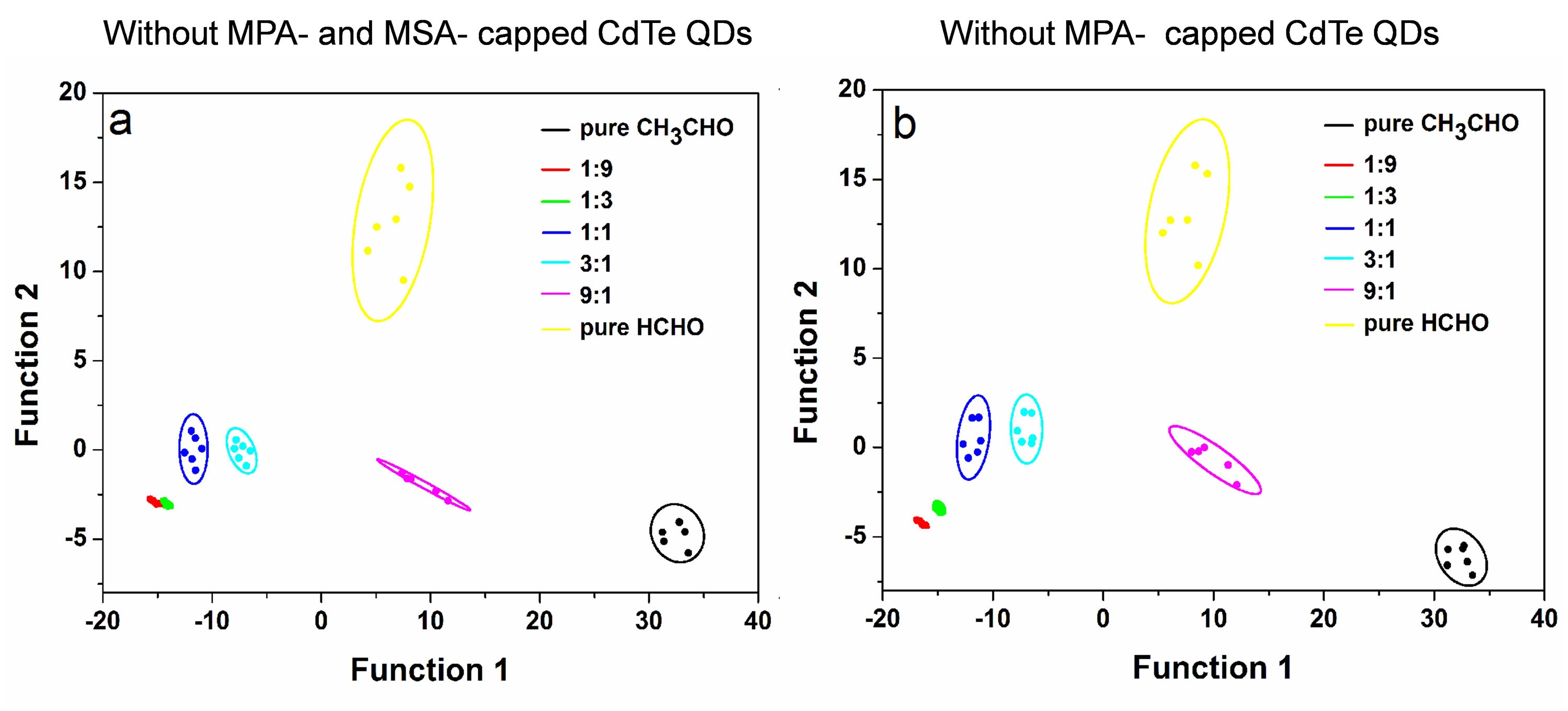


**Figure S11.** LDA cluster analysis for distinguishing different ratios of formaldehyde to acetaldehyde (total concentration of formaldehyde and acetaldehyde: 20 ppm, the ratios were for acetaldehyde/formaldehyde: v/v).

**Table S4.** Analytical results for formldehyde in real samples by the proposed method.

| **Samples** | **UV (mg/m^3^)^a^** | **This method (mg/m^3^)** |
| --- | --- | --- |
| 1 | 0.29 ± 0.01 | 0.33 ± 0.06 |
| 2 | 0.15 ± 0.01 | 0.21 ± 0.07 |
| 3 | 0.38 ± 0.01 | 0.32 ± 0.15 |
| 4 | 0.43 ± 0.01 | 0.48 ± 0.06 |
| 5 | 0.46 ± 0.01 | 0.42 ± 0.09 |

^a^Average ± standard deviation (n=3).

**Table S5.** Analytical results for formaldehyde in certified reference samples by the proposed method

| **Samples** | **Certified value^a^** | **Found value** |
| --- | --- | --- |
| BW3450 (mg/mL) | 10.1 ± 0.03 | 10.4 ± 0.11 |
| GBW(E)081701(μg/mL) | 104 ± 0.05 | 115 ± 0.21 |

^a^Average ± standard deviation (n=3).

**Table S6.** Visual analysis of real indoor air samples with the proposed sensor array (full pattern).


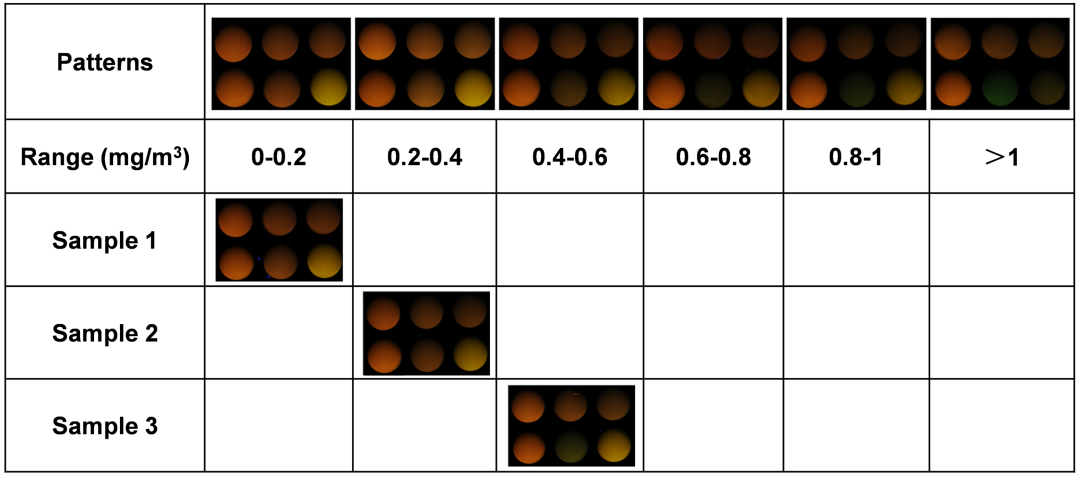


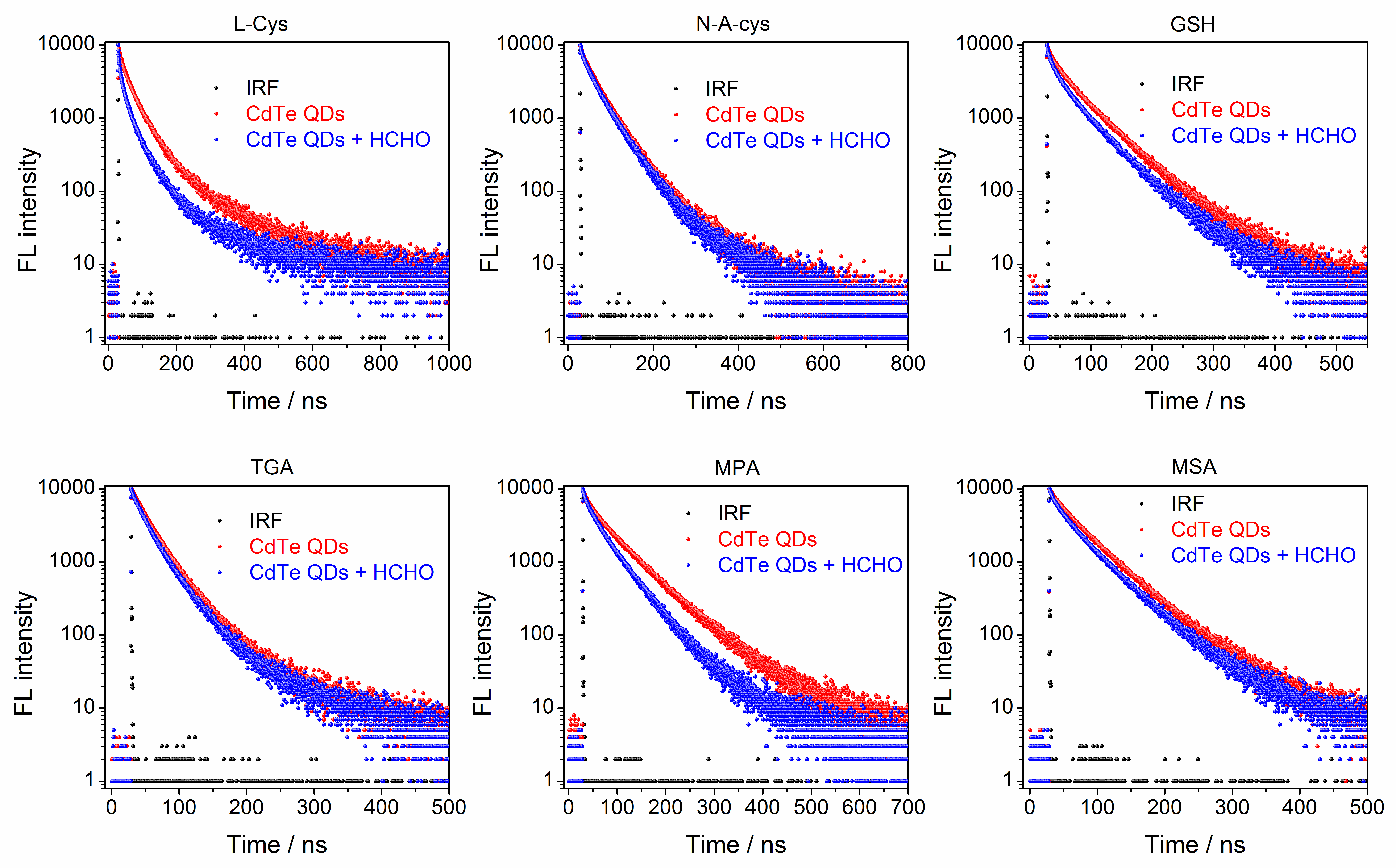


**Figure S12.** Fluorescence lifetime analysis of CdTe QDs in the absence and presence of formaldehyde.

**Table S7**. Frontier molecular orbitals of the different ligands capped CdTe QDs and their products after reaction with formaldehyde

| **Ligand (L)** | **(CdTe)_6_-L** | | **(CdTe)_6_-L-P_1_** | |
| --- | --- | --- | --- | --- |
|  | **HOMO** | **LUMO** | **HOMO** | **LUMO** |
| **L-Cys** | 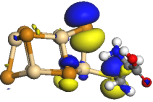 | 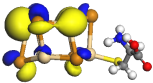 | 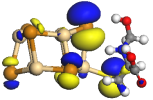 | 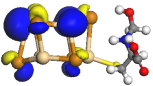 |
| **N-A-cys** | 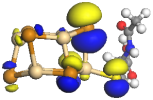 | 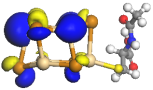 | 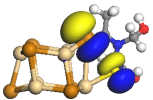 | 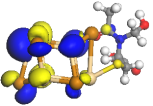 |
| **GSH_a_** | 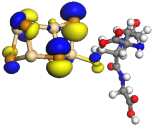 | 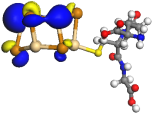 | 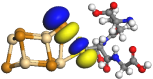 | 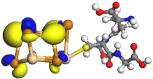 |
| **GSH_b_** |  |  | 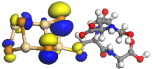 | 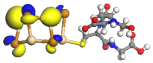 |

The calculated frontier molecular orbitals of (CdTe)_6_-L, (CdTe)_6_-L-P are depicted in Table S7**.** HOMO/LUMO reflects the electron distribution and the intermolecular charge-transfer character of the species. As shown in Table S7, all the HOMO-LUMO orbitals of three ligands capped with (CdTe)_6_ undergo change to a certain extent after reaction with formaldehyde.

**References**

[S1] Yu, W. W.; Qu, L. H.; Guo, W. Z.; Peng, X. G. Experimental determination of the extinction coefficient of CdTe, CdSe, and CdS nanocrystals. ***Chem. Mater.***, 2003, 15, 2854-2860.

[S1] Kuznetsov, A. E., Balamurugan, D., Skourtis, S. S. & Beratan, D. N. Structural and Electronic Properties of Bare and Capped CdnSen/CdnTen Nanoparticles (n = 6, 9). *J. Phys. Chem. C* **116 (12)** 6817-6830 (2012).

[S2] Bhattacharya, S. K. & Kshirsagar, A. Ab initio calculations of structural and electronic properties of CdTe clusters. *Phys. Rev. B* **75 (3)** 035402 (2007).
